# Drug Delivery Strategies and Nanozyme Technologies to Overcome Limitations for Targeting Oxidative Stress in Osteoarthritis

**DOI:** 10.3390/ph16071044

**Published:** 2023-07-23

**Authors:** Jessica Lee Aldrich, Arjun Panicker, Robert Ovalle, Blanka Sharma

**Affiliations:** J. Crayton Pruitt Family Department of Biomedical Engineering, University of Florida, Gainesville, FL 32611, USA; jessica.aldrich@ufl.edu (J.L.A.);

**Keywords:** nanozyme, osteoarthritis, reactive oxygen species, antioxidant, oxidative stress

## Abstract

Oxidative stress is an important, but elusive, therapeutic target for osteoarthritis (OA). Antioxidant strategies that target oxidative stress through the elimination of reactive oxygen species (ROS) have been widely evaluated for OA but are limited by the physiological characteristics of the joint. Current hallmarks in antioxidant treatment strategies include poor bioavailability, poor stability, and poor retention in the joint. For example, oral intake of exogenous antioxidants has limited access to the joint space, and intra-articular injections require frequent dosing to provide therapeutic effects. Advancements in ROS-scavenging nanomaterials, also known as nanozymes, leverage bioactive material properties to improve delivery and retention. Material properties of nanozymes can be tuned to overcome physiological barriers in the knee. However, the clinical application of these nanozymes is still limited, and studies to understand their utility in treating OA are still in their infancy. The objective of this review is to evaluate current antioxidant treatment strategies and the development of nanozymes as a potential alternative to conventional small molecules and enzymes.

## 1. Introduction

Osteoarthritis (OA) is a degenerative disease of the whole joint, characterized by cartilage loss and crosstalk with the bone, synovium, tendon, and nervous system [1,2,3]. Despite decades of efforts, there are no commercially available disease-modifying osteoarthritic drugs (DMOADS). Disease modification refers to the slowing or mitigation of structural changes to the joint, such as cartilage protection, in concert with improvements in pain and joint function. The current standard of care for OA is palliative management until disease severity dictates the need for a total joint replacement. This results in nearly 800,000 total knee arthroplasties each year in the United States [4]. As the average age of patients diagnosed with OA decreases due to traumatic knee injuries (post-traumatic osteoarthritis, PTOA) from sports, accidents, or military service, joint replacement is not a reasonable option. It is necessary to further understand the barriers to treating osteoarthritis and engineer therapeutic strategies to protect against disease progression.

Oxidative stress is a key driver in OA pathogenesis, resulting from the overproduction of reactive oxygen species (ROS) in the joint. The development of oxidative stress, either following a traumatic joint injury or due to aging-related joint changes, creates a negative signaling cascade which includes mitochondrial dysfunction, cartilage breakdown, synovial inflammation, and other downstream effects that are known contributors to OA [5]. While not the only contributing factor in the development of OA, oxidative stress is a strong candidate for therapeutic intervention. Oxidative stress occurs when there is an imbalance between the production of ROS and antioxidants within the joint [6,7]. Antioxidant enzymes are critical for scavenging excess ROS and maintaining redox balance—when these endogenous enzymes are overwhelmed, oxidative stress ensues, followed by oxidation of macromolecules and adverse secondary signaling [6,7]. There are currently no approved methods for mitigating oxidative stress in OA, though it remains an important disease target. 

The objective of this review is to identify and discuss current applications of ROS-scavenging nanomaterials, or nanozymes, to target oxidative stress in OA joints. This includes evaluating the limitations of current antioxidant-based therapies and the potential benefits of engineered material strategies. While treatment strategies for OA have been widely discussed and reviewed, this is the first comprehensive analysis of nanozyme technologies specifically for use in knee OA. 

### Important Considerations for Nanomaterial Delivery

Nanomedicine has been widely used in applications ranging from cancer [8], neurological disorders [8], and vaccine development [9], to name a few. Nanomaterials have been developing rapidly, owing to their potential use as both treatment tools and diagnostic tools [10,11]. These types of therapies can be engineered for targeted, site-specific delivery, delayed/controlled release, and novel, patient-specific treatments. In many clinical applications, nanomaterials have served as key enabling technologies for improving bioavailability and stability of small molecule drugs, proteins, and nucleic acids, though challenges with site-specific targeting often remain.

The use of nanomaterials to overcome the limitations specific to treating OA has been a rapidly advancing area of research and there have been many advancements in the field. It is becoming increasingly recognized that delivery of therapeutics to specific cells and tissues and in response to specific disease stimuli may be important. This also requires consideration of the unique transport barriers to overcome as well as disease-related mechanisms to leverage. For example, leveraging electrostatic interactions between a negatively charged cartilage matrix and a positively charged nanomaterial may improve cartilage targeting and retention [12,13,14]. Alternatively, stimuli-sensitive nanomaterials may be designed to respond to changes in enzyme function, pH, temperature, and endogenous oxygen levels and provide a targeted therapeutic approach, thereby improving efficacy and decreasing off-target adverse effects [15]. These are just a few of the many considerations necessary in designing nanomaterials to treat OA. A variety of other biomaterials engineered for intraarticular delivery include micelles [16], dendrimers [17], liposomes, and many others, have been thoroughly reviewed [18]. We point the reader to reviews focused on nanomaterial engineering for targeted delivery to the joint [14,19,20,21,22,23]. This review will focus on the emerging application of nanozymes as a potential treatment for OA. 

## 2. Understanding Antioxidant Strategies

The role of oxidative stress on disease progression in OA has been widely reviewed and characterized [5,24,25,26,27]. Subsequently, several exogenous antioxidants and antioxidant-like strategies have been studied for use as OA therapies [28,29,30]. The application of antioxidant-based therapies is an effort to maintain the necessary redox balance for healthy cell signaling without causing negative downstream effects. 

The relationship between ROS and antioxidants, in both function and location, is complex and interconnected. Cells must maintain a normal level of ROS to complete necessary cell signaling pathways, a complete loss of ROS may be just as detrimental to joint health as an overabundance [31]. It is also necessary to consider intra and extracellular locations of ROS and antioxidants. ROS targeting strategies will only be as effective as their ability to reach the necessary compartments where the ROS are produced; without this connection there may be a mismatch between the therapy location and the target. 

Specifically in OA, prolonged ROS overproduction that outpaces endogenous antioxidant function is detrimental to joint health. This overproduction may be caused by abnormal mechanical stimuli, increased inflammation, and mitochondrial dysfunction [32]. Mitochondrial dysfunction, for example, is a known hallmark of intracellular ROS overproduction and could be one key target for slowing disease progression; however, it may require antioxidant treatments tailored for mitochondrial targeting [33]. Alternatively, regulating the NF-κB signaling pathway or the NOX4 signaling pathway whose components may be found in the cytosol or inner mitochondrial membrane, respectively, may be other compartment-specific targets for antioxidant treatment in OA [34,35]. The numerous locations and interconnected nature of ROS/antioxidant relationships yield a variety of targets for antioxidant-based treatment. 

**Figure 1:**
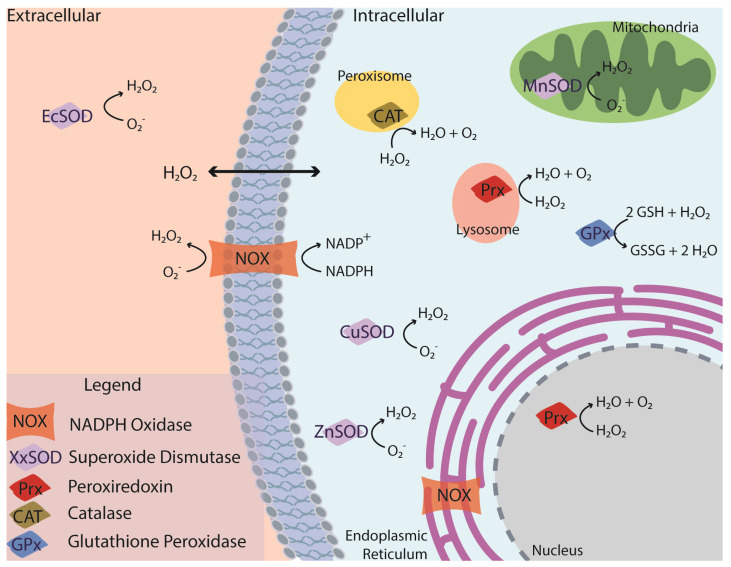
Key enzyme functions in the cartilage microenvironment that scavenge and produce ROS critical for intracellular signaling and chondrocyte function. Enzymatic functions occurring in both intra and extracellular regions validate the need to understand where treatments localize, to fully understand and evaluate therapeutic efficacy.

Reactive oxygen species implicated in OA include oxygen radicals (O_2_^−^, OH^−^) and nonradical, oxygen-containing reactive agents (H_2_O_2_, O_2_, O_3_, etc.) (Figure 1) [7,36]. Hydrogen peroxide (H_2_O_2_) is abundantly produced in OA and can cross the cell membrane, and thus is present in both intra- and extracellular microenvironments. H_2_O_2_ is a product of several antioxidant enzymes (superoxide dismutase (SOD) and NADPH oxidase (NOX)) and reacts with transition metals to yield a hydroxyl radical (-OH) [36]. In addition to the direct formation of radicals, H_2_O_2_ overproduction can disrupt the NF-κB signaling pathway and stimulate pro-inflammatory cytokines and chemokines that lead to cartilage degeneration [34,37]. Superoxide radicals (O_2_^−^) and nitric oxide radicals (NO) are present within the cell and react together to form peroxynitrite, a potentially cytotoxic ROS [38]. Superoxide anions are predominantly produced in the mitochondria and can be mediated by the mitochondrial electron transport chain [7]. These anions contribute to the production of H_2_O_2_ following a reaction with SOD. Hydroxyl radicals are present within a defined cellular compartment and can be produced by a reaction between O_2_^−^ and H_2_O_2_ or following a Fenton reaction [39]. The Fenton reaction is an oxidation process that causes the production of hydroxyl radicals from hydrogen peroxide and iron [40]. 

Glutathione, another key player in the antioxidant system, is predominantly reduced via glutathione peroxidase (GPX) in the cytosol and is retained within the cell, though GPX can be found in all cellular compartments. Similarly, specific forms of superoxide dismutase (SOD) are found in the extracellular space (EcSOD) while others can be found in the mitochondria (MnSOD) or the cytosol (ZnSOD, CuSOD). The different types of SOD use different transition metals as cofactors in the generation of H_2_O_2_ from oxygen radicals. The complexity and inter-connected nature of ROS generation has been well described in other reviews [38,39].

Antioxidant enzymes are predominantly found intracellularly; however, extracellular enzyme function is important in regulating cell–cell communication. These enzymes target specific ROS based on their location, as described above, and their mechanism of action. Transition metal ions, including iron and copper, are also found in the extracellular space and can produce H_2_O_2_ in the presence of OH^−^ [36] but they have also been shown to produce additional hydroxyl radicals [41]. Iron, specifically, may participate in the Fenton reaction, producing hydroxide and a hydroxyl radical when reacted with H_2_O_2_. Other intracellular antioxidants include GPX and peroxiredoxins. Peroxiredoxins (PRX), specifically in OA tissues, can be found within the mitochondria, nucleus, lysosomes, and cytosol [42]. PRXs protect against oxidative stress by reducing hydroperoxide and peroxynitrite. They have also been shown to play a role in the development of age-related OA [32,43]. GPX disposes of H_2_O_2_ through the oxidation of reduced glutathione (GSH) to oxidized glutathione (GSSG) is essential for maintaining redox homeostasis [7,44].

This vast network of enzymes, ROS, and cell functions makes it necessary to understand the timing and location of ROS overproduction, as it relates to developing oxidative stress. If antioxidant-like treatments cannot reach the site(s) of ROS generation, then the treatment cannot perform its intended function, thereby limiting therapeutic efficacy. Interestingly, few antioxidant-based therapies evaluate or address the issue of cellular and subcellular localization. Antioxidant strategies that consider and control ROS levels in different cellular and extracellular compartments may be necessary to achieve therapeutic success.

## 3. Current Antioxidant Strategies

Targeting oxidative stress is a promising treatment strategy for OA and other diseases caused by disruptions in redox signaling. In OA, translation of these therapies has been limited due to poor bioavailability and poor retention in the joint space. Current research has predominantly focused on oral intake of vitamins or nutraceuticals, but this approach is limited by poor stability and bioavailability to the joint [45]. Intra-articular injections of small molecules have similar limitations due to physical barriers in the joint. Targeting ROS production in the cartilage is difficult because the tissue is avascular and has a dense extracellular matrix (ECM), creating a unique transport barrier. The underlying subchondral bone, which exhibits abnormal remodeling during OA progression, is also challenging to access via the joint space [46]. Tissue localization of therapies can be hindered by rapid drug clearance through the synovial fluid via blood vessels in the synovium, thereby limiting the retention of treatments in the joint space. Once inside the joint, barriers to intracellular localization pose an additional challenge. Physiological barriers in the OA joint limit the effectiveness of several routes of administration for promising OA therapies. The addition of engineered multi-enzymatic treatment systems are a promising strategy to overcome these limitations.

Advances in the development of antioxidant mimicking biomaterial systems may overcome limitations of current therapeutic treatments. These challenges point to the reality, as described by Halliwell, that which ROS, how much, and the precise molecular target are key factors to consider in the development of therapies to tackle imbalance in any of these species [47]. In addition, further advancements in redox biology are necessary to measure ROS in vitro and in vivo are currently being developed. A critical limitation in this space is the inability to accurately, and sensitively, measure ROS produced within the system [39,48,49]. Due to the short half-lives of most ROS, they can be difficult to measure in vitro and in vivo. Although H_2_O_2_ is one of the most stable ROS, and is commonly produced in oxidative stress environments, it often requires sophisticated probes [49,50] or must be present in high quantities to be measured. This, along with challenges in targeting other ROS that are overproduced in OA, showcases a need for innovative strategies.

### 3.1. Overview of the Antioxidants Used for Osteoarthritis Therapy

Endogenous antioxidant enzymes are the most efficient mechanism to manage oxidative stress within the joint. However, in oxidative stress conditions, the supplementation with exogenous antioxidants, their mimics, and activators may be needed to restore redox balance and mitigate downstream disease pathways. Current antioxidant therapies under investigation include small molecules such as resveratrol, curcumin, and vitamins C, D, and E (Table 1). While promising, each of these antioxidants has unique challenges that limit their therapeutic efficacy. 

#### 3.1.1. Vitamins, Minerals, and Flavonoids

Natural, food-based compounds have been widely cited as a possible therapeutic strategy for treating OA [51]. Specifically, vitamins C, D, and E have been evaluated through clinical trials and other elements found in trace quantities through the body are becoming more popular. Vitamins C (L-ascorbic acid) and E (α-tocopherol) neutralize free radicals in aqueous phases and lipid membranes, respectively, but do so less efficiently relative to endogenous antioxidant enzymes. Vitamin C provides an electron to neutralize free radicals and is known to produce the semidehydroascorbate radical that is responsible for limiting the production of additional free radicals produced by endogenous enzymes [36]. Vitamin C is known to play a crucial role in promoting musculoskeletal development and is a cofactor for enzymes necessary for collagen matrix synthesis [7]. In a comprehensive review of vitamin C as an OA therapy, Dunlap et al. found conflicting results for pre-clinical and clinical trials of vitamin C supplementation. While the therapeutic effects of vitamin C supplementation are unclear, there is a consensus that overdosing vitamin C is detrimental to both the development and progression of OA [52]. In comparison, vitamins D and E, both membrane-bound antioxidants, have been investigated as potential OA therapeutics. Vitamin E has shown promising chondroprotective effects in rodent models of OA. However, Vitamin D, which is known for its role in regulating bone metabolism, is more well known for contributing to OA progression when it is deficient in the body [53,54,55,56]. Vitamin D supplementation has shown conflicting results on joint structure and OA-related pain [53,57]. Like vitamins C and E, oral supplements of vitamin D may not produce a disease-modifying effect. Additionally, most studies that involve oral super dosing of vitamins have had less than promising results in human studies [47].

Zinc, an essential nutrient for enzymatic functions and immune support, has been identified as another potential treatment for OA. Supplementation of zinc could block inflammation-induced oxidative stress [58]. The antioxidant-like function of zinc centers around the activation of Nrf2 which provides support for enzymes including SOD, GPX, and heme oxygenase-1. It may protect against oxidative stress and support the function of critical enzymes that maintain redox balance. In a rodent model of inflammatory OA, a daily supplement of 1.6 mg/kg/day of zinc was sufficient to decrease OA pathogenesis, specifically protecting cartilage structure, compared to a non-treated group. Dietary supplements, however, do have key limitations that inhibit their clinical translation. Specifically for zinc, it has been found that increasing doses does not increase therapeutic efficacy, and there is the potential for acute toxicity if overdosed. 

Quercetin, a flavonoid found in fruits and vegetables, has recently been studied as an alternative therapeutic for treating oxidative-stress-induced endoplasmic reticulum stress (ER stress). A known contributor to OA progression, ER stress plays a role in regulating chondrocyte apoptosis and cartilage degeneration [59]. Quercetin has chondroprotective effects including suppression of chondrocyte apoptosis and regulating SIRT1 (a key player in the biological response to oxidative stress). Through in vivo evaluation of quercetin given via intraperitoneal (IP) injection, it has been found that the compound attenuates cartilage degradation through activation of SIRT1 and AMPK [60,61].

Although the addition of supplemental antioxidants has been evaluated for several years, results surrounding disease modification are scarce and most therapies have stalled in pre-clinical models. As pure compounds, these strategies are ineffective for treating OA. However, the addition of adequate delivery vehicles may support their translation from pre-clinical models to clinical trials and commercial availability. 

#### 3.1.2. Resveratrol

Resveratrol is one of the most cited nutraceuticals for use in OA owing to its anti-inflammatory effects through inhibition of NF-κB [62,63]. Resveratrol produces antioxidant effects by scavenging free radicals [64]. It has also been suggested that resveratrol may compete with coenzyme Q to decrease ROS generation from the oxidative chain complex, scavenge O_2_^−^ radicals from mitochondria, and may inhibit lipid-peroxidation-induced byproducts from the Fenton reaction [65]. There is also evidence that resveratrol can maintain homeostatic levels of GSH in vitro [66]. Though these results in chondrocytes have yet to be confirmed, it is evident that the antioxidant functions of resveratrol require intracellular localization.

Resveratrol is predominantly administered via oral and injectable routes of delivery, with varying success in preclinical models [67]. Most therapies utilize free resveratrol that has not been encapsulated or integrated with other components [68,69,70,71]. Free drug treatments require injections as often as daily for 2 to 3 weeks [70,71] to maintain therapeutic levels within the joint. Currently, there is no evidence to support therapeutic residence within chondrocytes using these methods. And, while these dosing schemes may be effective in pre-clinical models, the translational ability of these therapies is limited due to the high dosing frequency and potential challenges with patient compliance. 

A limitation of free resveratrol is the risk of conformational change of the drug when exposed to microenvironments in the body. When studied in vitro, polyphenols such as resveratrol are dissolved in organic solvents before use, due to their low water solubility [72]. Organic solvents can artificially increase chondrocyte permeability and thereby increase the intracellular uptake of the polyphenol which does not recapitulate the joint space. 

To address some of these limitations, there have been advances in encapsulating or complexing resveratrol with biomaterials for improved drug delivery. For example, Cui et al. have coupled resveratrol with an oxidized cellulose aerogel (RLTA) creating a sustained release profile for the resveratrol [73,74]. The sustained release lasted for 5 h with a release of 40% total resveratrol encapsulated [74]. Application of the RLTA decreased inflammatory factors (IL-6, TNF-α) in an exercise-induced rodent model of OA. However, in vitro release and intracellular localization should be evaluated to advance this aerogel system. 

Alternatively, lipid-core nanocapsules (LNC) with a polycaprolactone biodegradable shell have been tested for combination resveratrol and curcumin delivery [72]. Co-encapsulation techniques such as the LNCs are a promising technique to deliver polyphenols that traditionally have limited stability profiles [75]. Resveratrol could be released for up to 24 h surpassing the retention time in other free drug models. As with the prior system, cellular and in vivo models with the LNCs will be necessary to further understand their efficacy as a therapeutic delivery system. 

In several aspects these strategies provide an improvement in free-drug methods of delivery. However, the LNC co-encapsulation system has not been used with chondrocytes or OA based applications [75]. The RLTA treatment, administered via oral gavage, showed promising effects on IL-6 and TNF-α expression [73]. This work indicates a potential for oral therapy, but further characterization is necessary to understand how it may be providing a disease modifying result. 

#### 3.1.3. Curcumin

Derived from turmeric, curcumin has been referred to as the “spice” for mitigating joint inflammation [76]. Curcumin is known to suppress the function of the NF-κB signal transduction pathway which can inhibit the cellular inflammatory response. It has also been shown to inhibit apoptosis by interrupting the expression of p38 and c-Jun N-terminal kinase (JNK). Lastly, curcumin acts as a free radical and H_2_O_2_ scavenger through H-atom donation from a phenolic group on the compound [77]. Therapeutic effects with curcumin may result from both intra and extracellular ROS scavenging, as it decreases general H_2_O_2_ production that may occur in both locations. Its effects on intra and extracellular ROS may enhance the therapeutic potential of curcumin and provide opportunities for various delivery vehicles or routes to be considered. 

Curcumin is limited by poor joint bioavailability, with oral dosing leading to bioavailability only as high as 1% [78]. However, oral supplementation remains the primary method of treatment evaluated for curcumin in OA. 

Similar to resveratrol, coupling the therapeutic effects of curcumin with materials to enhance bioavailability has expanded the therapeutic potential of this nutraceutical. For example, silk fibroin nanoparticles (SFN) loaded with curcumin improved ROS scavenging properties of curcumin, as measured by DPPH free radical scavenging in an acellular model, compared to free-drug. They also decreased the activity of markers connected with increased inflammation (RANTES, IL-6, NO) [79]. The SFNs created a controlled release of curcumin for as long as 24 h, which may overcome joint retention challenges. Further in vivo evaluation is necessary to determine if overcoming the limitation of joint retention is enough to make the therapeutic clinically viable, depending on the route of administration. The fact that the LNCs described previously improved curcumin release to 72 h supports efforts to apply sustained release systems to nutraceutical use [75].

Alternatively, a prodrug strategy, amphiphilic curcumin polymer micelles (ACP), using an acid-activatable curcumin polymer, has been developed to improve curcumin bioavailability in the joint. Kang et al. integrated curcumin with a polymer backbone such that the polymer can self-assemble into micelles in acidic environments [80]. The pH-responsive nature of the micelles creates a theranostic approach that can have both disease detection and therapeutic capabilities. In a neutral environment, the micelles are characterized by fluorescent quenching; however, in an acidic environment, the micelles dissociate, allowing the fluorescent particles to be visualized and the curcumin to be released in the joint. The ACP micelles, when given via an intra-muscular injection in an inflammatory model of OA, suppressed IL-1β and TNF-α expression and showed potential for cartilage protection. Micelles were injected every 3 days for a period of 28 days, and while the compounding anti-inflammatory effects may be beneficial in decreasing cartilage breakdown, it does not appear to overcome the barrier of therapeutic retention in the joint. 

Curcumin has long been evaluated as an approach to decrease oxidative stress and reduce inflammation through antioxidant functions that may support intra and extracellular management of ROS production [79]. Like resveratrol and other vitamin treatments, implementing the right delivery system for the desired route of administration will be necessary to advance and implement curcumin as a prescribed treatment for OA. 

#### 3.1.4. Exogenous Enzymes

Given their efficient ROS-scavenging properties, the delivery of exogenous antioxidant enzymes has been explored for the treatment of OA. Exogenous enzymes, specifically catalase and SOD, have been injected into the joint with varying success. 

The addition of exogenous SOD has been discussed since the 1980s, ranging from applications with liposomes or microinjections to intra-articular or systemic injections of the enzyme alone [81]. Some well-known challenges in using exogenous SOD to scavenge ROS include the inability of SOD to enter cells, its inability to survive unchanged in the GI tract, and that the byproducts of SOD must be less toxic than the superoxide that is broken down in the reaction to be effective as a therapeutic. Early results of SOD injection used 2–16 mg SOD injected on a weekly or biweekly basis and showed improvements in patient reported pain and joint function. However, radiographic evidence of joint structure remained unchanged based on the therapeutic treatment, indicating that the effect was only on the short-term inflammatory aspects of the disease. In studies with higher dosages of SOD (16 and 32 mg) given weekly for 3 weeks via IA injection, there was a reduction in OA symptoms for up to 3 months [82]. Limitations of using free enzymes may be overcome by loading SOD into nanoparticle systems. However, many of these systems exhibit poor enzyme loading, decreased enzymatic function after release, or enzyme inactivation following conjugation [83,84,85]. 

More recently, a polymersome made of amphiphilic synthetic coblock polymers was used in a pre-clinical model of OA [86]. Loaded with SOD, these nanoparticles were designed to breakdown superoxide radicals. When given via IA injection, SOD-NPs were retained in the joint for up to 4 weeks following a DMM surgery, an improvement on the retention time of most injectable therapies. Gui et al. found that the SOD-NPs localized to the synovium in vivo and that they decreased the ROS production in vitro [86]. This recent advancement in the use of SOD with a nanoparticle system shows that the application of exogenous enzymes may still be a viable option if paired with a suitable carrier. 

Though less commonly evaluated, catalase is another endogenous enzyme that could be used as a therapeutic agent for slowing the development of oxidative stress. Free catalase is limited by a short half-life within the joint [87]. Previous studies have attempted to overcome this limitation by PEG-conjugating the enzyme to improve biostability [88,89]. In a rodent model of OA, intra-articular injection of cationized catalase was retained in the joint and mitigated inflammation [90]. However, the addition of non-cationized exogenous catalase did not influence disease progression. This work also points to H_2_O_2_ concentration as the rate-limiting factor to catalase function. This dose-dependent relationship should be considered in future work with enzyme mimicking nanomaterials; however, it is rarely discussed in the current literature. Recent efforts to utilize catalase as a treatment for OA have focused on applications in rheumatoid arthritis [91]. Unfortunately, beyond challenges of joint stability and bioavailability, exogenous enzymes can be expensive and complex to produce, making translation into the clinic challenging [92]. 

#### 3.1.5. Superoxide Dismutase Mimics and Activators

SOD plays a significant role in redox homeostasis and the development of oxidative stress. In a homeostatic environment, SOD catalyzes the conversion of O_2_^−^ into oxygen and H_2_O_2_. Through this, SOD decreases one type of ROS, O_2_^−^, by creating another which can be more readily broken down—H_2_O_2_. To support endogenous SOD function, treatments that mimic SOD function or contribute to increased production of SOD have been developed and tested in pre-clinical models. SOD mimics have been studied across a variety of medical fields [91] with some applications in orthopedic research [93,94,95]. 

Manganese porphyrin has been used as a synthetic SOD mimic that scavenges O_2_^−^ in environments with oxidative stress. Manganese porphyrin has grown in clinical relevance as a potential cancer therapeutic [90,96] and is understood to protect against mitochondrial damage related to oxidative stress [97]. Although not yet used in OA models, manganese porphyrin has been studied on intervertebral disc cells to mitigate oxidative stress in degenerative disc disease [98]. When encapsulated in a chondroitin sulfate microparticle system, there was a sustained release of manganese over 3 months. Even more promisingly, the microparticles were taken up by nucleus pulposus cells. Further study into their SOD-mimicking functions and the potential for intracellular SOD function will be necessary to advance this therapy. Success in the disc model is promising and may be translatable into OA models. 

Another small molecule, BNTA (N-[2-bromo-4-(phenylsulfonyl)-3-thienyl]-2-chlorobenzamide), induces SOD expression and superoxide anion elimination [93]. The application of BNTA in vitro has been found to significantly increase extracellular SOD (EcSOD or SOD3) function and facilitate ECM production in OA chondrocytes, OA cartilage explants, and in a rodent model of OA. The application of BNTA following an ACLT (anterior cruciate ligament-transection) surgery resulted in decreased OA related joint changes compared to a vehicle control based on histological results. Similar to other injectable therapies, one key limitation in the application of BNTA as a DMOAD is the need to deliver an IA injection two times per week for a sustained duration (4 or 8 weeks, in this example) to see the desired effects. 

Mitigation of extracellular ROS could play a role in interrupting disease-related inter-cellular signaling within the extracellular matrix. BNTA has a unique ability to enhance the function of EcSOD, which could play a role in downstream ROS production and cellular signaling cascades. Both manganese porphyrin and BNTA showcase the importance of supporting enzyme function in both the intra- and extra-cellular microenvironment. They further advance the idea that therapies with cell-specific and compartment-specific targeting may be necessary to prevent or slow oxidative stress effectively, to produce disease-modifying effects. 

**Table 1:** A sampling of antioxidant therapeutics used for the treatment of OA across clinical and pre-clinical models.

Antioxidant Therapies Used in OA					
*Therapeutic*	*Antioxidant Effect*	*Treatment Method*	*Disease Model*	*Effects*	*Citation*
Resveratrol	↑ Intracellular SOD	50 mg/kg 3 days/week for 8 weeks	MIA-induced OA, male Wistar rats	↓ inflammatory cytokine expression, ↑ NRF2 expression, ↓ NF-κB	Wei 2018 [68]
Curcumin	↑ Phosphorylation and DNA binding activity of NRF2, free radical scavenger	Varied oral dosing regimens	Human OA patients, clinical trials	↓ NF-κB, ↓ chondrocyte apoptosis, ↓ oxidative stress	Chin 2016 [76]
Manganese porphyrin	SOD mimic, scavenges O_2_^−^, activates NRF2	BuOE MPs @ 100 μg/mL	Human nucleus pulposus cells	↓ radiation-induced oxidative damages, ↓ inflammation by blocking NF-κB activation	Lee 2022 [98]
BNTA	SOD mimic, ↑ SOD3, and ↓ O_2_^−^	IA injection of BNTA, 100 μL at 0.015, 0.15, 1.5 mg/kg 2x/week for 4/8 weeks	ACLT-induced OA, male Sprague Dawley rats	↑ expression of ECM components, ↑ cartilage ECM synthesis, ↓ inflammatory mediators	Shi 2019 [95]
Vitamin C	Scavenges free oxygen radicals	Varied oral dosing regimens	Human OA patients, clinical trials	↓ chondrocyte apoptosis, ↓ production of pro-inflammatory markers, overdose can be detrimental	Dunlap 2021 [52]
Zinc	↓ ROS production, ↑ GSH by MIA	1.6–8.0 mg zinc/kg/day via gavage for 2 weeks	MIA-induced OA, male Wistar rats	↓ arthritic progression, ↓ proteoglycan loss, ↓ IL-1β levels, ↑ antioxidative capacity	Huang 2018 [58]
Quercetin	↓ ER stress, ↑ SIRT1 and AMPK	50–100 mg/kg quercetin via IP injection daily for 12 weeks	MMT-induced OA, male Sprague Dawley rats	↓ chondrocyte apoptosis, ↓ cartilage degeneration	Feng 2019 [60]

Abbreviations: MIA—monoiodoacetate; ACLT—anterior cruciate ligament transection; MMT—medial meniscus transection.

### 3.2. Overview of Delivery Methods Used for Osteoarthritis Therapy

Although we are always told that taking vitamins and eating fruit were the tricks to increasing antioxidant levels in our bodies, the limited stability and absorption of antioxidants moving through the digestive system hinder their efficacy in diseases such as OA. Beyond ingesting these therapies, clinical trials have evaluated topical treatments with limited success, and most pre-clinical models have settled on high dose rates of therapies via frequent intraarticular (IA) injection. The route of administration for antioxidant-based therapies is a key consideration in their efficacy. 

#### 3.2.1. Oral Delivery

Ingestible antioxidants, such as resveratrol and curcumin, are the most common antioxidant strategies. Oral therapies can be implemented as a lifestyle choice or may be prescribed after disease diagnosis based on pain or radiographic evidence of OA. Unfortunately, these are often implemented after disease onset and may have limited efficacy on protecting the joint from further damage. Although popular in clinical trials for nutraceutical-based approaches, oral delivery can be associated with gastrointestinal (GI) complications or negative side effects [99]. Oral supplements may also have limited bioavailability and may be inactivated through the metabolic process. Drug delivery strategies for antioxidants must overcome formulation barriers, including low solubility and low stability, and biological barriers, particularly short GI transit time, low permeability/absorption, and pre-systemic clearance. 

Nutraceuticals are frequently given via oral supplementation through dietary changes [100]. For example, preclinical studies supplementing resveratrol in a high-fat diet with a mouse model of obesity-induced OA have shown joint structure recovery through histological evaluation and a reduction in chondrocyte apoptosis [101]. In a clinical trial, oral resveratrol administered once daily (500 mg) for 90 days increased serum levels of aggrecan which may suggest a protection or recovery of joint structure; however, there was no radiographic evidence of joint change [100]. However, serum levels of type II collagen, a necessary building block for cartilage, were unchanged. In another study, oral dosing of curcumin (500 mg 3x/day for 12 weeks) led to a reduction in OA-related pain [102]. Although promising, a critical limitation in translating oral applications of free-drug, beyond stability and bioavailability concerns, is patient compliance. There are no concerns about toxicity, but the dose rate may not be sustainable for long term treatment.

Oral delivery of vitamins C and E are another common home remedy that have been tested as a dietary supplement to slow or prevent joint breakdown. However, clinical trials have consistently shown no correlation between dietary changes with these vitamins and OA disease progression [54,103,104,105]. Willow bark extract, another supplement compound with aspirin-like anti-inflammatory and ROS-scavenging properties, has also been investigated for OA in preclinical models. In a rodent model of OA, this compound has been shown to improve cartilage health and reduce inflammatory mediators (TNF-α, IL-1β, and IL-6). However, in clinical trials, the results regarding pain management and disease-modifying effects have been mixed [106]. Currently, these antioxidants are available as over-the-counter supplements, but they have not been classified as DMOADs and are not considered a standard of care. 

#### 3.2.2. Topical Application

Topical applications of a therapeutic allow for targeted treatment at the site of pain and provide a patient centered approach, giving the patient control of applying the treatment. Most topical treatments include topical NSAIDs, which are often a first line of therapeutic treatment in age-related OA [107,108]. Topical application of NSAIDS, particularly diclofenac [109], for pain management have shown some benefit in early-stage OA [108]. However, this standard of treatment does not provide disease-modifying effects, specifically radiographic evidence of cartilage protection. 

Curcumin nanoparticles (NPs) applied to the skin at the site of pain have been shown to improve joint retention with sustained therapeutic effects when compared to oral supplementation of free curcumin [110]. Curcumin nanoparticles applied topically to the joint were measurable in the infrapatellar fat pad within 3 h of application; however, they were not present after 6 h. The curcumin NPs were not visible in the cartilage or synovium, indicating that they did not travel beyond the fat pad. Histological results have shown improved cartilage pathology following the treatment, indicating that curcumin in the fat pad may have joint wide effects. The pain relief and cartilage protection from the in vivo model may be attributed to the suppression of inflammatory cytokines measured in vitro. However, further studies to confirm these effects may be necessary. 

Though an interesting strategy, bioavailability to the joint is a key concern in the adoption of topical treatments. The mechanism by which therapies reach the desired joint tissue through the physical barriers, including the skin, musculature, and synovium, is currently unknown. Visible particle uptake in the fat pad is a promising step toward understanding these mechanisms in future work. If effective, topical strategies such as the curcumin nanoparticles may improve patient compliance.

#### 3.2.3. Parenteral Therapies

Most pre-clinical models rely on injectable therapies to target a site-specific dose either indirectly (systemic injections) or directly into the joint space (intra-articular injections). This strategy avoids some of the limitations of other routes such as poor bioavailability and poor joint targeting. 

##### Injectable Materials

The size and type of delivery vehicle and cargo are key considerations in the development of nano- and microparticles used for parenteral treatments. Microparticles, ranging from synthetic polymers to natural macromolecules, have been shown to improve drug stability and allow for the sustained release of drugs. Synthetic polymers, such as poly lactic-co-glycolic acid (PLGA), have been engineered into microspheres that can be loaded with a drug cargo [111]. These microspheres significantly prolong the residence time of drugs in the joint space and can have sustained release up to 3 months after injection [112,113]. Microparticles typically function as depot systems and are non-phagocytocable above 10 μm. Through diffusion and/or degradation, they release the therapy into the joint space over time, reducing the frequency of injections required. This may be useful for improving extracellular ROS scavenging or, if the released cargo can be taken up into cells, intracellular ROS scavenging. 

On a smaller scale, nanoparticles made from natural macromolecules such as chitosan, hyaluronic acid (HA), polyester amides, and other peptides or from synthetic polymers and molecules, including hollow mesoporous silica nanoparticles, gold nanoparticles, nanocrystal polymer particles, and metal–organic frameworks have been evaluated as potential OA therapies [114]. Nanoparticle systems can protect the antioxidant until it is delivered intracellularly, which may improve drug targeting for intracellular oxidative stress. Both, microspheres and nanoparticles also improve drug bioavailability and stability, allow for better sustained and controlled release of the drug, and require lower dosages than free-drug treatments. Microparticle formulations for other OA drugs have been investigated clinically, but injectable, antioxidant-based treatments have not expanded beyond preclinical models [11]. Nano and micro scale particle systems may also be an effective strategy in oral or topical routes of administration and may protect the antioxidant from metabolism and improve bioavailability. Currently, most particle formulations are being investigated as IA injectable therapies in pre-clinical models.

Similar to polymeric encapsulation, liposomes have also been used to provide targeted delivery to specific cell types [115,116]. Liposomes are spheres enclosed by a lipid bilayer, mimicking the cell membrane and allowing for more effective uptake into cells. Liposomes may contribute to joint lubrication, which may also contribute to OA treatment by supporting the function of lubricin and the synovial fluid [117,118,119,120,121]. As a drug delivery vehicle, liposomes have been evaluated for delivering various types of drugs—mostly non-steroidal anti-inflammatory drugs (NSAIDs), cartilage matrix components, and nucleic acids for gene therapy [122,123,124]. However, liposomes loaded with antioxidants have recently been evaluated as a method to prevent oxidative stress in OA [125]. Gold nanoparticles with antioxidant functions have been loaded into surface-active phospholipid mimetic (DPPC) liposomes as a therapeutic for a rodent model of OA. Gold NPs are known to down regulate Cox-2, IL-1β and PGE-2, which are implicated in disease pathogenesis. 

Injectable hydrogels are highly tunable and can also be used as a depot delivery system for therapeutics into the joint space [126]. Hydrogels for OA therapy have been investigated for their potential to deliver NSAIDS, hyaluronic acid, glycosaminoglycan, and antioxidant therapeutics. Injectable hydrogels with ROS-scavenging properties have been investigated as one solution for sustained release within the joint space. A hydrogel containing epigallocatechin-3-gallate (EGCG) has shown ROS-scavenging properties and protects against the upregulation of IL-1β [127,128]. Eicosapentaenoic acid, an antioxidant that scavenges free radicals [129], has been added to a gelatin-based hydrogel to evaluate its effects on slowing disease progression in a preclinical model of OA [130]. Creating a sustained release profile, on the order of weeks, this therapy is an improvement over most injectable therapies that are known to clear the joint within hours.

Injected therapeutics are typically cleared from the joint through lymphatic draining and in the capillaries that underlie the synovium. Fine tuning the drug delivery system to provide the right drug at a sufficient dose and time within the joint is important to consider when developing injectable therapies. 

##### Injection Methods

Intraperitoneal (IP) injections provide a route of administration that bypasses metabolism in the digestive system and are often used in pre-clinical rodent models. Free curcumin administered via an IP injection has been shown to reduce the progression of OA by inhibiting apoptotic pathways and proinflammatory mediators and cytokines in rats (50–150 mg/kg) [131] and mice (50 μM) [132]. Although effective, these studies required high dose rates and had clear dose-dependent chondroprotective effects. Alternatively, acid-activatable micelles delivered via intramuscular injection may improve the bioavailability of curcumin [80]. Using a prodrug form of curcumin encapsulated in the micelle, the therapeutic could bypass physical barriers for intracellular delivery with a sustained drug release profile. When injected, these micelles improved chondroprotective effects compared to free drug while using a lower dose rate (2.5 mg/kg, 5 mg/kg, respectively). However, they are a promising strategy to utilize a common nutraceutical therapeutic in a delivery vehicle that can overcome traditional OA barriers. Systemic injections face similar challenges as other indirect routes of administration—due to the barriers associated with ensuring targeted therapies enter the joint to provide optimal antioxidant effects. 

Intra-articular (IA) injections are the most direct method for targeted therapies to enter the joint space. They ensure the delivery of the therapeutic compound into the joint and may improve the bioavailability of the therapy while limiting the potential off-target effects. They also provide opportunities to improve stability, retention, and sustained-release characteristics using delivery vehicles loaded with antioxidant-based therapies. A critical limitation of IA injections is the need for frequent, high-dose injections (i.e., 2–5 times/week for the duration of the study) to obtain therapeutic benefit [114,133]. Frequent dosing schemes are the result of poor joint retention, with injected therapies often cleared from the joint within a few hours. 

IA injections are a standard practice in pre-clinical models of OA, although strategies involving antioxidant-like or ROS-scavenging therapies have limited examples. Direct injection may overcome limitations of other routes of administration including bioavailability and retention challenges. However, there may still be concerns about rapid clearance and poor localization to specific target tissues within the joint space. 

### 3.3. Engineering Strategies for a Biological Challenge

The studies reviewed so far highlight the potential for antioxidant therapies to treat OA; however, they require high dosing and frequent injections, which are problematic for preclinical-to-clinical translation. One emerging strategy involves the use of synthetic nanomaterials with inherent antioxidant properties. Often referred to as “nanozymes,” these materials demonstrate ROS-scavenging kinetics that approach that of endogenous antioxidant enzymes, while being able to address the issues of stability, bioavailability, and cost of delivering exogenous antioxidant enzymes [134,135]. These new strategies have been widely used in energy transfer and environmental engineering applications [136], and have recently demonstrated potential to provide a multi-enzyme-mimicking approach in biomedical applications, including OA treatment.

## 4. Biomaterial Strategies as Antioxidant Mimics to Treat OA

Nanozymes are a class of biomaterials that are characterized by their ability to display enzyme-like characteristics [134,137,138]. These biomaterials have grown in prevalence since the early 2000s; however, their exact definition is still up for debate. 

In the context of this work, we evaluate nanomaterials with at least one known enzyme-mimicking function. One limitation in evaluating and characterizing nanozymes is that the mechanism of action for the enzyme-mimicking function may not be fully understood. Further exploration of these novel materials may shed light on these comparisons; however in this case we will consider any enzyme-like activity to be worth discussing in this context. 

Nanozymes have emerged as an enzyme-mimicking solution to the high cost and poor stability of endogenous enzymes. Biomaterials as enzyme-mimics can be broadly characterized by their specific enzyme function: catalase (CAT), SOD, peroxidase, and oxidase mimics [139,140]. Medical interest in nanozymes has been increasing [135,141,142], for example, with cobalt ferrite nanozymes [143] and single-atom manganese oxide [144] used in cancer applications [142]. They have also been used in applications for treating inflammatory bowel disease [145] and as biosensing platforms [137,146]. While nanozymes and their derivatives can be found widely in the literature, direct applications of antioxidant-mimicking therapies for osteoarthritis are limited (Table 2). Manganese dioxide and ceria oxide, used alone or in concert with other strategies are the most cited nanozyme strategies for OA applications. 

Some benefits and considerations for nanozymes include the potential for compartment-specific uptake, as with other therapies discussed here, which may be important in mitigating the oxidative stress. Prolonged extracellular retention may also have therapeutic benefits, given the relatively fewer endogenous mechanisms in the EC space. The possibility to engineer nanomaterial properties to direct their tissue and cell localization and antioxidant functions is an advantage over conventional antioxidant systems. Additionally, multi-enzyme mimicry may provide therapeutic benefits that are not specific to a single target, which could be advantageous in OA. 

### 4.1. Metal Oxides

Metal oxide nanoparticles are the most commonly studied nanozyme for OA [48]. Metal oxides are highly tunable, can be combined with a variety of other treatments, and are cost-effective to produce. It is expected that the smaller the particle size, the stronger the enzymatic activity and the more readily they can be taken up within the cell [55]. Metal oxide NPs smaller than 8 nm may enter the cell via direct diffusion [48]. Larger metal oxide NPs may enter the cell through endocytic mechanisms, such as clathrin or caveolin-mediated endocytosis, after adhering to the cell membrane. 

Despite the innate benefits of using metal oxide nanozymes, some common limitations must also be addressed. Depending on the specific metal oxide structure and composition, there may be concerns of intracellular toxicity or inactivity of the therapeutic based on the cellular compartment to which they localize. Metal oxides may generate additional free radicals, for example, through the Fenton reaction where partially or fully oxidized nanoparticle systems may generate additional ROS [158]. Iron- and cerium-based NPs have been known participate in this reaction and develop additional free radicals, potentially limiting their efficacy. 

Common metal oxides include cerium oxide (CeO_2_) [159], manganese oxide (MnO) [149,160,161], magnesium oxide [162], and zinc oxide [58,162].

#### 4.1.1. Cerium Oxide 

Cerium oxide NPs, also known as ceria oxide or nanoceria, can have SOD-, CAT-, oxidase-, and peroxidase-mimicking activities depending on their formulation [163,164,165,166]. Ceria nanoparticles have been shown to scavenge H_2_O_2_ in vitro [147], provide chondroprotection in vivo [148], and ameliorate inflammation in models of rheumatoid arthritis [166]. Cerium oxide nanoparticles produce their enzyme mimicking effects while fluctuating their valance states between Ce^3+^ and Ce^4+^ [164]. The NPs exhibit more SOD-like activity during the conversion from Ce^3+^ to Ce^4+^ and have more CAT-like activity when converting from Ce^4+^ to Ce^3+^ [48]. Size also plays a critical role in their oxidative function, with smaller nanoparticles exhibiting stronger SOD-like effects than larger particle systems [164]. 

In a rodent model of temporomandibular joint (TMJ) osteoarthritis, nanoceria (nCe) preserved cartilage and subchondral bone structures [148]. Three days after the induction of TMJ OA, nanoceria particles were injected into the joint cavity and effects were observed after 10 days. A single injection of 20 nm particles ranging from 100–2000 μg/mL appeared to preserve cartilage and subchondral bone structure. Dashnyam et al. also found that the nCe supported macrophage polarization toward the M2, or anti-inflammatory, phenotype by suppressing the pro-inflammatory effects of the disease. They also exhibited ROS-scavenging effects in a model of chondrocytes exposed to H_2_O_2_ followed by treatment with nCe. The measurements for ROS production and scavenging focused on changes in intracellular ROS; while there was no determination that the nCe were taken up by the cells, the decrease in ROS is indicative of effective antioxidant functions. The advancement of a single-dose therapeutic is a promising improvement compared to other direct injection strategies that require consistent dosing to show a therapeutic effect. Future applications of this therapy may be dependent on the duration of the therapeutic effect, but the inhibition of ROS production may be enough to create a sustained effect. Other joints affected by OA, specifically the knee, may need further evaluation to determine whether the barriers within the joint inhibit the function of nCe. 

When used in combination with HA, cerium oxide nanoparticles improved therapeutic efficacy and may be a viable strategy for improving the application of other well-known therapeutic agents that are limited by bioavailability and targeting strategy challenges [147]. The interaction between the HA and CeO_2_ NPs, as well as their location within the cell, are currently unknown. However, when used in combination, they protect Col1A1 expression in the presence of H_2_O_2_, a downstream effect of oxidative stress. The system also protects against characteristic loss of proteoglycan when exposed to H_2_O_2_. 

The predominant function of cerium oxide NPs relies on their ability to target intracellular ROS, therefore requiring them to be designed for intracellular localization. However, cerium oxide is non-degradable and drug clearance may be a critical limitation in implementing this nanosystem as an OA therapeutic [167]. Evaluation of nanoparticle localization, as well as the safety and efficacy of these therapies are necessary steps to advance cerium oxide as a potential OA strategy.

#### 4.1.2. Manganese Oxide

Manganese oxide has been used in biomedical and environmental applications [48]. It has been shown that various formulations of MnO have the potential to mimic peroxidase, SOD, and CAT functions [150,160]. There is also some evidence that MnO nanozymes may improve neuropathic pain responses in rodent models of OA [168]. The structure of manganese oxide nanosystems affects the catalytic activity and stability/degradation rates of the material [169]. This structure–function relationship is important for understanding MnO material function and its relevance in treating OA; however, it has not been widely studied. 

Manganese is an essential nutrient that is necessary for biological functions [170]. It acts as a cofactor for a variety of enzymes including arginase, glutamine synthase, and MnSOD. The byproducts of Mn based nanoparticles create Mn ions that support the function of these enzymes and may improve mitochondrial functions to scavenge ROS production. The potential overabundance of intracellular Mn needs to be mitigated, as too much may cause further mitochondrial dysfunction and oxidative stress. Additionally, there is some risk of Mn toxicity, specifically in the brain [171]. However, this risk is likely minimal due to the low dose used in most studies of manganese oxide treatments for OA. 

Manganese oxide NPs are characterized by their ability to catalyze the breakdown of H_2_O_2_ into water and oxygen, providing SOD-, peroxidase-, and potentially, catalase-like functions in vitro. PEG-stabilized MnO_2_ has been used as a stand-alone therapeutic treatment [149] to scavenge H_2_O_2_ and decrease inflammatory markers relevant to osteoarthritis. Kumar et al. engineered MnO_2_ NPs with a cationic zeta potential and size of less than 13 nm to leverage electrostatic interactions and integrate with the dense extracellular matrix [149]. It is expected that the MnO_2_ NPs are also small enough to be taken up intracellularly and may be able to scavenge H_2_O_2_ produced within the cell, though this has yet to be reported. They have also shown chondroprotective effects in cartilage explants, with a decrease in glycosaminoglycan loss and nitric oxide production following IL-1β stimulation. Via cartilage targeting, these NPs have also shown improved joint retention on the order of days. Combining antioxidant effects with chondroprotection and increased joint retention make MnO_2_ NPs a promising therapeutic for OA treatment. Additional evaluation of in vivo effects will be necessary to further the application of this nanozyme therapy. 

Chen et al. utilized a hollow-MnO_2_ (h-MnO_2_) that may protect against cartilage damage in a surgical model of OA. In this model, h-MnO_2_ NPs decreased the presence of inflammatory mediators (IL-1β, IL6) in serum and may prevent joint remodeling [151]. Similar to Kumar et al. [149,151], the h-MnO_2_ NPs were coated with poly(allylamine) hydrochloride (PAH) to functionalize the surface of the hollow MnO_2_ NPs [151]. In mice, 30 μg/mL h-MnO_2_ NPs given 3x/week for 4 weeks showed protection of the cartilage and joint structure with a surgical model of OA. This dosing scheme is consistent with the high dose rates found with other injectable-based therapies which may, like the others, be a limitation for the long-term application of this therapy. The results suggest that the h-MnO_2_ protected against characteristic cartilage breakdown and bone remodeling consistent with the DMM model and is a promising option for future work. 

Other methods utilizing MnO_2_ as a therapeutic have leveraged combination treatment strategies. Zhou et al. fabricated an MnO_2_ nanozyme-encapsulated hydrogel by dispersing bovine serum albumin (BSA)-MnO_2_ nanoparticles in a hyaluronic acid/platelet rich plasma (PRP) gel network [152]. This conglomerate is intended to provide multi-functional support to cartilage. MnO_2_ NPs, as previously discussed, can slow the development of oxidative stress through CAT- and SOD-like functions. The MnO_2_ NPs specifically target H_2_O_2_ but may have broader antioxidative effects that can be leveraged in this system. Additionally, PRP promotes chondrocyte proliferation and has been widely used as a potential treatment for joint injuries that allows for patient-specific treatment. The multifunctional properties of this hydrogel system are aimed at targeting specific challenges in treating the OA joint: sustained release of the therapy for up to 3 days, decreasing ROS production in the presence of the hydrogel, and providing structural protection to the joint following an in vivo inflammatory response. Overcoming some of the limitations of a single nanozyme treatment, this hydrogel also utilizes components found naturally within the joint. As with many of the other therapies discussed here, there is a need to do further research on the behavioral effects of this therapy and a need to better understand intracellular mechanisms of action to fully understand how the therapy is protecting the joint. 

Carboxymethyl-chitosan-assisted MnO_x_ (WY-CMC-MnOx) nanoparticles leverage the theranostic properties of MnO nanozymes in combination with a cartilage-targeting peptide as a treatment for the early stages of osteoarthritis [153]. Manganese oxide is a known potential contrast agent and has been used as a theranostic agent in tumor-based cancers [172]. Early diagnosis of OA through MR imaging may provide additional opportunities to interfere with the negative feedback loop of oxidative stress that promotes OA pathogenesis [153], and is a practical application of MnO_x_ for orthopedics. Lin et al. found that the WY-CMC-MnO_x_ particles improved MR imaging of cartilage lesions, owing to their small size and cartilage targeting properties. In models with mesenchymal stem cells, the WY-CMC-MnO_x_ promoted chondrogenesis and protected against cartilage breakdown in vivo. Complexing components with chondroprotective or therapeutic effects may provide an advantage for treating OA by improving retention in the joint without re-engineering the basic material properties of the therapy.

Presently, there are only four known applications of Mn-based particle systems for managing oxidative stress production in OA. However, outside of the OA field, research has evaluated encapsulated MnO_2_ [173] and MnO_2_ used to decorate the surface of larger particle systems [174] as techniques to scavenge ROS from oxidative stress environments. Both particle systems were developed at the micro-particle scale and may have limited ability to translate as an OA therapy but showcase the versatility of MnO_2_ as a possible treatment for oxidative stress. As a transition metal conjugate, MnO_2_ may provide therapeutic effects both intra and extracellularly, catalyzing the breakdown of H_2_O_2_ into water and oxygen through CAT-mimicking functions or by decomposing H_2_O_2_ into OH^−^ if the Mn^2+^ ion dissociates from the particle in the extracellular matrix [36]. Further understanding of the intracellular localization of these therapies will be necessary to understand their full therapeutic potential. 

#### 4.1.3. Iron Oxides

Iron oxide nanomaterials have a broad range of functions owing to the combination of superparamagnetic functions and enzyme-mimicking functions. Iron oxide particles, referred to as IONzymes, have the potential to act as a theranostic nanozyme [141]. Ferromagnetic nanoparticles (Fe_3_O_4_) possess peroxidase-like activity, behaving like horseradish peroxidase (HRP) [175], and catalyzing the oxidation of standard peroxidase substrates such as TMB and DAB. Additionally, the magnetic properties of Fe_3_O_4_ provide opportunities to capture the particles after use. IONzymes follow Michalis–Menten kinetics, and both Fe_2_O_3_ and Fe_3_O_4_ have been reported to have peroxidase- and catalase-like functions. Gold-coated, superparamagnetic iron oxide nanoparticles leverage the properties of both IONzymes and gold, thereby acting as a multi-functional treatment strategy. These nanoparticles decreased inflammation in a collagenase-induced model of OA [176]. By leveraging these characteristics, further described by Gao 2007, iron oxide nanomaterials have therapeutic potential as a nanozyme for the treatment and detection of osteoarthritis [175]. However, iron-based materials may contribute to the production of additional ROS through the Fenton reaction, which may limit their therapeutic efficacy. Iron-oxide-based nanomaterials have been used broadly in the OA field as a mechanism to label and track cell and treatment movement in vitro and in pre-clinical models [177,178,179,180,181]. However, these studies do not address the potential enzyme functions of this material. Future work with other iron oxide systems may reveal additional multi-functional treatment systems effective for treating OA. 

#### 4.1.4. Additional Metal Oxides

There is an interest in evaluating a broad spectrum of biocompatible metals as potential nanozymes due to their promise as a treatment for oxidative stress. To date, biological evaluation has been conducted for silver-, aluminum-, cadmium-, copper-, magnesium-, titanium-, vanadium-, tungsten-, and zinc-based oxide particle systems [162]. Zhang et al. conducted a proteome-wide assessment of oxidative stress responses to metal oxide nanoparticles with human macrophages. Macrophages are critical immune cells in OA progression that contribute to the inflammatory state of the joint. They evaluated 11 different nanoparticles, including ceria-based NPs which have been previously discussed. From these 11 particles, there was a correlation between the level of proteome change, the induction of ROS-responsive proteins, and the eventual cytotoxic outcome following particle exposure. Taken from their results, it may be beneficial to evaluate the ROS-scavenging properties of metal oxides with low toxicity and minimal proteome change, such as MgO or SiO_2_, in a model of osteoarthritis. Additionally, this work showed that ceria oxide nanoparticles may also be useful in treating oxidative stress without causing off-target downstream effects. Luo et al. propose the potential use of magnesium oxide nanoparticles, due to their therapeutic potential as an ROS-scavenging agent [182], and zinc oxide due to its ability to inhibit gene expression of inflammatory markers and the effects of exogenous zinc [58]. While the list of promising therapeutics is continuing to evolve, only a few metal oxide nanozymes have been evaluated for the treatment of osteoarthritis, lagging behind the implementation and evaluation of nanozymes in other applications.

### 4.2. Prussian Blue Nanozymes

Prussian blue nanozymes are another advancement in engineered nanosystems with catalytic functions. Prussian blue is a versatile and biocompatible material known for its distinct blue color [183]. The compound, Fe_4_[Fe(CN)_6_]_3_, derives many of its properties from charge transfers through across Fe atoms and has been cited as a potential theranostic in biomedical applications. With a variety of uses in medicine, Prussian blue is best known for its function as an antidote for cesium and thallium poisoning [184]. Approved by the US Food and Drug Administration, Prussian blue nanozymes are known to have robust antioxidative-mimicking properties. Specifically, they leverage electron transport properties to exhibit peroxidase-, catalase-, SOD-, and other ROS-scavenging functions. Prussian blue nanozymes are highly tunable, with sizes ranging from ultrasmall (~3.4 nm) to >100 nm in hydrodynamic diameter based on the synthesis method. They are cost-effective to produce and do not generate additional free radicals typically found in other iron-based particle systems through the Fenton reaction. Because these particle systems can be engineered with a variety of different catalytic properties, it will be important to assess their compartment-specific localization within cells and their ability to be endocytosed in order to understand which ROS are being targeted and how the therapeutic effect is being achieved. 

Hollow Prussian blue nanozymes (HPBzymes) are one formulation that has been engineered for use in osteoarthritis [154]. HPBzymes were designed with a mesoporous structure to increase the specific surface area, with a size of 78 nm and a zeta potential of 1.85 mV. The pores of the mesoporous surface area range from 2–5 nm and have improved the reactions between HPBzymes and the substrates within the OA microenvironment. In vitro models have showcased the potential therapeutic effects of the HPBzymes, catalyzing the breakdown of OH^−^, OOH, and H_2_O_2_. In a rat MMT model of OA, the application of the HPBzymes (1x/week for 4 weeks) significantly reduced the structural effects of the MMT surgery (via histological analysis). Immunofluorescence has also shown a decrease in the expression of iNOS and COX-2. A high dose of HPBzymes (3.6 μg/mL) was more effective than a low dose (0.36 μg/mL). And, with injections 1x/week the use of the HPBzymes may be an improvement over many other injectable therapies given more frequently. Compartment-specific localization may still need to be addressed with this nanosystem, in large part due to their size and neutral charge. 

Another Prussian-blue-based system leverages a pH-responsive, biodegradable structure (HMPBzymes) [155]. The HMPBzymes measure 210 nm in hydrodynamic diameter with a zeta potential of –20 mV and are readily taken up by bone-marrow-derived macrophages, indicating the likelihood of ROS scavenging from within the cell. Macrophages have also shown the ability to polarize from an M1, proinflammatory, phenotype to an M2, anti-inflammatory, phenotype. This may indicate a shift in the joint microenvironment to promote a decrease in inflammatory cytokines and other downstream mediators of oxidative stress. A key contribution of this particle system is the ability to inhibit hypoxia as measured by the suppression of HIF-1α and the generation of oxygen in vitro. When given via IA injection following an inflammatory model of OA, weekly injections of the HMPBzymes have been shown to significantly improve joint structure. The HMPBzymes were also retained within the joint space for at least 7 days, protecting against a complete loss of therapeutic effects between injections. This advancement is important in overcoming the limitations of joint retention and sustained therapeutic effects. And, as one of few studies focused on macrophage function in OA, this work has shown the ability to have a therapeutic effect on joint structure without targeting cartilage.

Various forms of HPBzymes have been widely studied for use in other disease conditions such as ischemic stroke [185], inflammatory bowel disease [186], neurological decline [187], osteoporosis [188], and cancer [189]. Their advancement across a broad range of fields is a valuable step toward the application of HPBzymes in clinical trials and their potential availability in the future. 

### 4.3. Gold-Based Nanozymes

Gold-based, micro- and nano-scale therapies are another therapeutic intervention that has have been tested in OA applications, but they are more commonly utilized in rheumatoid arthritis applications [190,191,192]. Gold-based nanotherapies used in OA applications range from biologic carriers [193], a use as miRNA biomarkers [194], as a standalone therapy [157,195], and being integrated with iron oxide nanoparticles to combine antioxidant functions [176]. Despite their wide use, little is known about the antioxidant properties of gold-based therapies. Some studies suggest that gold-based treatments have peroxidase-mimicking functions which can help downregulate pro-inflammatory responses [196], or that they may support the production and function of catalase [197]. However, most studies do not discuss the potential antioxidant properties of this biomaterial. The translation of gold NPs into clinical settings is, in part, limited by issues of aggregation in the kidneys, liver, and spleen [198,199], and they have been shown to induce the formation of ROS, thereby having the opposite effect to we would expect [200]. These negative side effects have yet to be evaluated in OA applications, but are important to consider with the development of this treatment as a potential OA therapy. As the antioxidant effects of gold NPs have not been widely accepted, only a few of the current applications of these therapies to OA have been evaluated here. 

As a potential OA therapeutic, gold NPs offer several benefits including a high degree of tunability, and they are easy to combine with other biologic components. Filho et al. synthesized a 20-nm gold NP (GNP) that was given in combination with HA (0.9%) via IA injection in Wistar rats, following an MMT surgical procedure [156]. They found that the combination of GNPs and HA protected cartilage structure and decreased the pro-inflammatory cytokines. The GNP + HA treatment also decreased the effects of oxidative stress including nitrite and myeloperoxidase production, to name a few. It is expected that the GNPs improved integration of the HA therapy with the local joint environment. In terms of antioxidant function, this system was found to support the activation of NRF2, which activates KEAP1, leading to an increased production of antioxidants. However, the details of this pathway are uncertain. This is a promising therapeutic approach combining a nanozyme material with a commonly used adjuvant therapy, and future work may support its translation into a clinical setting. 

Similarly, Abdel-Aziz et al. used a similar gold nanoparticle (AuNP) with a small size (20 nm) and positive charge but coupled its application with Diacerein [157]. Diacerein is an anthraquinone derivative that inhibits IL-1β and has been recommended by the European League Against Rheumatism since the early 2000s [201]. Although typically used as an RA treatment, Diacerein used in combination with the AuNPs had promising effects related to slowing the progression of OA [157]. When given orally, the AuNPs + Diacerein improved oxidative stress markers, decreased DNA fragmentation, and protected cartilage structure more effectively compared to the MIA-induced OA controls. These results also indicated that the AuNPs were safe for use and did not have toxicity concerns commonly attributed to gold-based therapies. Interestingly, this is one of two studies highlighted in this review that utilized female rodents, which speaks to the need to evaluate sex as a biological variable in OA therapies. Further evaluation of the function of these therapies and a deeper understanding of their ROS functions will be necessary to further this application. 

There may be promise in furthering our understanding and use of gold-based nanomaterials for OA. However, a deep evaluation of their antioxidant-like properties will be necessary to advance their application as a nanozyme in any field. 

### 4.4. Single-Atom Nanozymes 

Single-atom catalysts have been developed with a variety of different metal active sites to create catalytically active nanomaterials [144,202]. An SAzyme with a Cl-Cu-N_4_ active center has been designed with identical geometric and electronic structures as naturally occurring SOD [202]. These SAzymes were designed to scavenge O_2_^−^ radicals and were able to degrade H_2_O_2_ in a catalase-mimicking function. The aggregation of SAzymes in chondrocytes has been shown to decrease the levels of intracellular ROS. This suggests that the SAzymes can enter chondrocytes and support endogenous enzyme function. In vivo, these effects were consistent as the SAzymes protected against cartilage breakdown following an ACLT model of OA. While further investigation of this therapy is necessary, it remains a promising and novel approach to enzyme-mimicking strategies that may overcome many of the limitations of current therapies. 

### 4.5. Additional Considerations in the Development of Nanozyme Technologies 

There is a significant number of considerations when designing nanomaterials as treatments or delivery vehicles for OA. The nanozymes presented here have only been evaluated in vitro and in rodent preclinical models (Table 2). However, molecular transport at these scales may not translate at larger scales, such as with large animal models or humans. Transport properties change with increasing cartilage thickness [13,14,203,204], meaning that therapies developed to target cartilage may encounter differences in tissue retention as the thickness increases. Once in the cartilage, if the nanomaterial is intended to target intracellular localization to chondrocytes, transport through the cartilage ECM, the pericellular matrix [205,206], and the cell membrane should be taken into account [19,207,208]. Upon entry into the joint, nanomaterials will develop a protein corona, a protein coat that attaches the material surface which can influence nanomaterial interactions with cells and tissues [196,209,210]. These considerations, as well as the proper delivery method and route of administration, may change depending on the specific location of interest. Numerous reviews on these nanoparticle characteristics and others necessary for joint delivery have been published [19,20,21,99,210].

In addition, nanomaterial fate and byproducts should be considered in the design and application of nanozymes. As ROS production differs intra- and extracellularly, nanozyme function should be matched with its intended location within the joint. Nanomaterial systems too large to passively cross cell membranes may be endocytosed and degraded in lysosomes or trafficked/exocytosed from the cell. Alternatively, systems that bypass these fates and remain in the cytosol could impact intracellular ROS either in the cytosol, mitochondria, or the nucleus. Over time, byproducts of these materials may produce neutral species or additional free radicals. While the exact byproducts are not known for all nanozymes, it is important to consider how they may affect the system and could further exacerbate the imbalance of antioxidants to ROS. Similar to discussions on nanomaterial transport, nanozyme fate may differ between pre-clinical rodent models of OA and clinical studies due to the scale of cartilage structure. 

Endogenous enzymes are the most efficient mechanism for scavenging ROS and maintaining redox balance in the joint. The rate at which enzyme-mimicking strategies are deployed in the joint and the amount of therapeutic benefit is also important to consider in their design and implementation. Augmenting endogenous enzymes, the addition of synthetic nanomaterials (or nanozymes), could support enzymatic function without disrupting cell function. Although most of our focus has been on the intracellular effects of nanozymes, the actual location of most nanozymes after delivery has not been widely studied. Future work evaluating the intracellular localization and antioxidant pathways of nanozymes will be critical in advancing their efficacy. 

## 5. Conclusions

At the time of writing, a literature search for ‘Nanozymes’ and ‘Osteoarthritis’ results in 11 results from as early as 2020. While nanozymes may be widely used in other applications, their use in orthopedics is in its infancy. Early adopters to the development and use of these materials may be on the forefront of a potential technology that could improve our ability to target oxidative stress in OA, and consequently improve treatment for the disease. However, a common limitation of the therapies discussed here is the limited understanding of how the antioxidant mechanisms work, and for how long. As this field develops, it will be critical to consider and understand the ROS target for which these nanozyme systems are developed and how their antioxidant functions improve therapeutic efficacy. 

Nanozymes may play a role in the continued development of other cutting-edge therapies, specifically for personalized medicine and gene therapy applications. Characteristics of nanozymes, similar to other nanomaterials, can be tailored through simple biomaterial and synthesis changes. Nanozymes can also be loaded into scaffolds or gels to provide additional benefits in inflammatory tissues. This flexibility and the enzyme functions of nanozymes make them a compelling tool for more advanced applications. Future work with gene therapy and tissue-engineered materials may leverage these properties. 

The development of nanozymes specifically for OA is still in its infancy. This class of therapies may overcome the limitations of traditional treatments that have often stalled following pre-clinical evaluation. Many of the challenges in developing DMOADs, such as the ability to identify and treat the disease before cartilage loss, will continue to be a challenge for these nanomaterials. However, engineering a treatment system that mimics endogenous functions is a promising gateway to further advancements in the OA field. It is possible that additional therapies and formulations of nanozymes will become a key focus for treating OA over the next several years. 

## Figures and Tables

**Table 2:** Nanozyme therapies currently being investigated as ROS scavenging treatments for OA.

Nanozymes in OA					
***Nanozyme***	*Antioxidant Function*	*Treatment Method*	*Disease Model*	*Effects*	*Citation*
**HA-CeO_2_**	SOD Mimic, ↓ H_2_O_2_ concentration	Coincubation with 0.2 g/mL CeO_2_ NPs for 1–3 days	0.3 mM H_2_O_2_ for 30 min. on bovine chondrocytes	↓ cartilage degeneration, ↓ chondrocyte apoptosis, ↑ H_2_O_2_ scavenging	Lin 2020 [147]
**Nanoceria (nCe)**	SOD Mimic	30 μL 250–2000 μg/mL nCe injected into synovial joint cavity	CFA-induced TMJ OA, male Sprague Dawley rats	↓ cellular apoptosis, ↓ catabolic proteins and pro-inflammatory cytokines (IL-1β/TNFα), ↑ polarization of M2 macrophages, ↑ anti-inflammatory cytokines (IL-10) and chondrogenic glycoproteins	Dashnyam 2021 [148]
**MnO_2_**	Scavenges H_2_O_2_, catalase mimic	20 μL 5 mg/mL via IA injection	Healthy male Lewis rats	↓ loss of glycosaminoglycans, ↓nitric oxide, ↓ H_2_O_2_	Kumar 2019 [149]
**MnO_2_**	Scavenges H_2_O_2_, catalase mimic	0–200 μg/mL	Murine insulinoma cells (BTC3)	↓ oxidative stress, mimics SOD and CAT, ↓ cell death	Tootoonchi 2017 [150]
**H-MnO_2_**	Scavenges H_2_O_2_	0.2 mL 30 μg/mL H-MnO2, 3x/week, 4 weeks, via IA injection	DMM-induced OA, female C57BL/6 mice	↓ cartilage degeneration, ↓ subchondral bone remodeling, ↓ inflammatory cytokines (IL6, IL1β, TNFα)	Chen 2021 [151]
**HA/PRP/BM hydrogel**	ROS scavenging	1.5 mg/mL HA/PRP/BM hydrogel, single injection 3 days after MIA induction	MIA-induced OA, Sprague Dawley rats	↓ cartilage degeneration, ↓ subchondral bone remodeling	Zhou 2022 [152]
**WY-CMC-MnOx**	↓ oxidative stress	100 μL 2.5 mg/kg MnOx, every 2 weeks for 6 weeks via IA injection	DMM-induced OA, male Sprague Dawley rats	Theranostic agent, ↑ chondrogenesis, ↓ cartilage degeneration	Lin 2022 [153]
**Hollow Prussian-blue nanozymes (HPBzymes)**	Scavenges H_2_O_2_, OH^−^, OOH	0.36–3.6 μg/mL HPBzyme injection 1x/week, for 4 weeks post surgery	MMT-induced OA, male Sprague Dawley rats	↓ IL1B, ↓ Rac1-ROS-NFκB signaling pathway	Hou 2021 [154]
**Hollow manganese Prussian-blue nanozymes (HMPBzymes)**	Catalyzes H_2_O_2_ into O_2_, ↓ HIF-1α expression, ↓ ROS levels	80 ug/mL HMPBzyme 1x/week for 4 weeks	MIA-induced OA, male Sprague Dawley rats	↓ inflammation, polarizes macrophages from M1 to M2	Xiong 2022 [155]
**Gold Nanoparticles with Hyaluronic Acid (GNPs + HA)**	↑ NRF2 to induce signaling of genes that encode antioxidants	2.5 mg/L GNP +/− 0.9% HA injection every 15 days for 90 days	Median meniscectomy induced OA, male Wistar rats	↑ NRF2, ↓ inflammation, ↓ cartilage degeneration	Filho 2021 [156]
**AuNPs**	↓ ROS production	30 ug/kg AuNPs +/− Diacerin (50 mg/kg) via oral route using a stomach tube weekly for 5 weeks	MIA-induced OA, female Sprague Dawley rats	Preserved GPX and SOD function in kidney and liver, ↓ cartilage degeneration, ↓ DNA fragmentation	Abdel-Aziz 2021 [157]

Abbreviations: CFA—complete Freund’s Adjuvent; TMJ—temporomandibular joint; DMM—destabilization of medial meniscus; MIA—monoiodoacetate; MMT—medial meniscus transection.

## Data Availability

Data is contained within the article.

## Abbreviations

ACLT	Anterior cruciate ligament transection
ACP	Amphiphilic curcumin polymer micelles
AuNP	Gold nanoparticles
BNTA	(N-[2-bromo-4-(phenylsulfonyl)-3-thienyl]-2-chlorobenzamide)
BSA	Bovine serum albumin
CAT	Catalase
CeO_2_	Cerium oxide
CFA	Complete Freund’s adjuvant
DMM	Destabilization of medial meniscus
DMOAD	Disease-modifying osteoarthritis drug
ECM	Extracellular matrix
ER	Endoplasmic reticulum
GI	Gastrointestinal
GNP	Gold nanoparticles
GPX	Glutathione peroxidase
GSH	Glutathione
GSSG	Oxidized glutathione
h-MnO_2_	Hollow manganese dioxide
H_2_O_2_	Hydrogen peroxide
HA	Hyaluronic acid
HPBzymes	Hollow Prussian blue nanozymes
HRP	Horse radish peroxidase
IA	Intra-articular
IP	Intraperitoneal
LNC	Lipid core nanocapsules
M2	Macrophage
MIA	Mono-iodoacetate
MMT	Medial meniscus transection
MnO_2_	Manganese dioxide
nCe	Nanoceria
NO	Nitric oxide
NOX	NADPH-oxidase
NP	Nanoparticle
NSAID	Nonsteroidal anti-inflammatory drug
OA	Osteoarthritis
PAH	Poly(allylamine) hydrochloride
PEG	Polyethylene glycol
PLGA	Poly-lactic co glycolic acid
PRP	Platelet-rich plasma
PRX	Peroxidredoxin
PTOA	Post-traumatic osteoarthritis
RLTA	Oxidized cellulose aerogel
ROS	Reactive oxygen species
SAzymes	Single-atom nanozymes
SFN	Silk fibroin nanoparticle
SOD	Superoxide dismutase
TMJ	Temporomandibular joint
